# Supplementary material for: Genome-Wide Novel Genic Microsatellite Marker Resource Development and Validation for Genetic Diversity and Population Structure Analysis of Banana
Source: Genes (Basel). 2020 Dec 9;11(12):1479. doi: 10.3390/genes11121479 (PMC7763637; doi:10.3390/genes11121479)
Supplement: Supplementary file 1 [file genes-11-01479-s001.pdf]

Genome-wide novel genic microsatellite marker resource development and validation for genetic diversity and population structure analysis of Banana

Manosh Kumar Biswas, Mita Bagchi, Dhiman Biswas, Jennifer Ann Harikrishna, Yuxuan Liu, Chunyu Li, Ou Sheng, Christoph Mayer Ganjun Yi and Guiming Deng

Table S1. Summary of transcript sequences assembly.

Sequence Data	Count Transcript Sequences	SSR containing Sequences	% of SSR containing transcriptome
Musa acuminata	45891	22223	48
Musa balbisiana	33074	15902	48
Musa itinerans	32456	9986	31
Musa schizocarpa	32784	11558	35
EST	77919	9047	12
Total	222124	68716	31

Table S2. Distribution of different types of SSR within UTR and CDS (%).

		Compound	Di	Tri	Tetra	Penta	Hexa	AT rich	AT/GC Balance	GC rich	Class I	Class II
Count	3UTR	437	6008	3115	335	80	79	4731	3843	1525	4866	5233
	5UTR	539	7294	3338	362	71	78	5584	4460	1674	5665	6053
	CDS	585	7418	4584	375	97	108	6230	4420	2573	6272	6951
%	3UTR	1.24	17.10	8.87	0.95	0.23	0.22	13.46	10.94	4.34	13.85	14.89
	5UTR	1.53	20.76	9.50	1.03	0.20	0.22	15.89	12.69	4.76	16.12	17.23
	CDS	1.66	21.11	13.05	1.07	0.28	0.31	17.73	12.58	7.32	17.85	19.78

Table S3. FRSMs annotated by GO category .

Item	No. of FRSMs
Annotated with all three GO categories	3891
Annotated with two GO categories	4148
Annotated with only one GO categories	3407

Table S4. Distribution of unique FRSMs among three GO categories.

GO categories	Number of FRSMs	Number of unique FRSMs
BP (biological process)	29446	670
CC (cellular component)	20734	1692
MF(molecular function)	14272	1045
**Overall number of unique elements**		**3407**

Table S5. Comparative mapping between *M. acuminata* (A genome) and Foxtail millet.

	**Chromosome**	Foxtail millet									
		F01	F02	F03	F04	F05	F06	F07	F08	F09	Total
***M. acuminata* (A genome)**	**A01**	1	4	10	2	0	0	3	2	2	24
	**A02**	0	0	2	3	0	2	3	2	1	13
	**A03**	4	3	3	0	4	3	3	1	4	25
	**A04**	3	2	9	5	0	3	5	0	6	33
	**A05**	1	6	6	2	2	0	1	2	5	25
	**A06**	1	2	4	4	3	0	3	5	1	23
	**A07**	0	5	3	3	2	0	2	2	1	18
	**A08**	2	1	4	6	4	2	2	3	8	32
	**A09**	1	2	1	3	2	2	0	1	2	14
	**A10**	1	0	2	6	2	0	3	3	1	18
	**A11**	1	1	3	1	1	2	0	1	5	15
	**Total**	15	26	47	35	20	14	25	22	36	240

Table S6. Comparative mapping between *M. acuminata* (A genome) and Rice.

	**Chromosome**	**Rice**												
		**R01**	**R02**	**R03**	**R04**	**R05**	**R06**	**R07**	**R08**	**R09**	**R10**	**R11**	**R12**	**Total**
***M. acuminata* (A genome)**	**A01**	13	3	4	5	1	1	1	1	0	0	0	2	31
	**A02**	5	2	2	3	0	0	3	1	2	1	1	0	20
	**A03**	5	5	5	3	2	1	4	1	1	2	1	1	31
	**A04**	13	4	4	0	1	1	0	2	1	0	2	0	28
	**A05**	5	2	2	2	1	2	2	0	1	0	2	1	20
	**A06**	3	2	6	2	2	4	2	1	2	2	2	2	30
	**A07**	6	1	1	1	4	1	2	1	0	0	2	1	20
	**A08**	8	2	6	3	2	1	1	2	2	1	1	1	30
	**A09**	3	3	1	2	3	3	4	1	1	1	1	2	25
	**A10**	3	4	1	2	0	3	0	0	1	1	1	1	17
	**A11**	3	1	2	1	1	1	1	0	0	0	0	2	12
	**Total**	67	29	34	24	17	18	20	10	11	8	13	13	264

Table S7. Comparative mapping between *M. acuminata* (A genome) genome and Sorghum.

	Chromosome	Sorghum										
		**Sor01**	**Sor02**	**Sor03**	**Sor04**	**Sor05**	**Sor06**	**Sor07**	**Sor08**	**Sor09**	**Sor10**	**Total**
M. acuminata (A genome)	**A01**	11	3	2	1	2	3	1	3	0	1	27
	**A02**	6	2	5	5	0	0	0	0	1	2	21
	**A03**	5	9	1	0	3	4	3	4	3	1	33
	**A04**	7	7	4	2	2	3	3	5	0	1	34
	**A05**	4	3	1	1	2	4	3	1	0	1	20
	**A06**	4	6	5	1	3	2	0	2	1	4	28
	**A07**	3	2	5	1	0	5	3	0	1	1	21
	**A08**	6	2	0	3	6	2	3	1	3	0	26
	**A09**	3	3	1	1	0	2	2	3	0	2	17
	**A10**	5	4	1	3	4	3	0	3	1	2	26
	**A11**	2	2	2	4	0	2	1	0	1	1	15
	**Total**	56	43	27	22	22	30	19	22	11	16	268

Table S8. Percentages of the orthologous regions from the targeted species having SSR motifs.

Genome	Number of Mapped FRSMs	No of orthologous region having SSR repeats	% of orthologous region having SSR repeats
M. acuminata (A genome)	17561	14692	83.66
M. balbisiana (B genome)	15373	14332	93.23
M. schizocarpa (S genome)	16286	14975	91.95
Foxtail millet	240	13	5.45
Rice	264	19	7.32
Sorghum	268	10	3.79

Table S9. Wet lab assay summary.

	Count	%
Total number of FRSMs marker tested	273	
No. of markers amplified	259	95
No. of polymorphic markers	203	74
No. of mono morphic markers	46	17
No. of non-scorable markers	24	9
No. of markers produces a single allele	249	91
No. of markers produces multiple locus	24	9
No. of markers produces fragments that are either longer or shorter than the expected size	36	13
No. of markers transferable to Musa relatives	194	71
Total allele count	715	
Allele range	2-12	
Average No. of alleles per locus	3.5	

Table S10. Percent transferability of FRSM from different *Musa* Spp.

Sl. No.	Genomic Group	[1]% of transferability	
		In silico	Wet lab
**1**	*M. acuminate* (AA)	44	74
2	*M. balbisiana* (BB)	31	-
3	*M. schizocarpa*	33	-
4	GY37 (AAB)	-	73
5	GY160 (ABB)	-	71
6	GY112 (AAAB)	-	68
7	GY60 (AAA)	-	75
8	GY109 (AAAA)	-	72
9	GY70 (ABBB)	-	66
10	BGY3 (BB)	-	61
11	GY127 (AABB)	-	69
	Overall		71

[1] iMarkers were considered as transferable if resulting in a clearly amplified target sized product in at least 3 of the tested genotypes

Table S11. Summary of genic variation statistics for all Loci.

Primer ID	Locus	Sample Size	na*	ne*	I*	PIC #	Major.Allele.Frquency#	Availability #
C01P3AA00057	G002	50	3	2.61	1.03	0.51	0.55	0.92
C01P3AA00134	G004	50	3	2.94	1.09	0.58	0.38	0.80
C01P6AA002298	G008	50	6	3.44	1.44	0.66	0.46	0.98
C01P3AA002665	G009	50	3	2.31	0.91	0.47	0.55	0.92
C01P2AA005299	G021	50	4	3.15	1.24	0.59	0.52	0.84
A2M001208	G030	50	3	2.04	0.74	0.39	0.53	0.94
A2M000167	G037	50	3	2.18	0.91	0.35	0.76	0.80
AB2M006344	G040	50	3	2.04	0.74	0.38	0.62	0.78
AB2M006866	G041	50	3	2.02	0.84	0.26	0.84	0.76
AB2M002966	G052	50	4	2.69	1.14	0.54	0.55	0.94
NovelTSSR000395	G063	50	3	2.17	0.75	0.53	0.45	0.81
NovelTSSR000641	G075	50	4	2.78	0.89	0.57	0.55	0.82
NovelTSSR000760	G084	50	2	2.04	0.79	0.64	0.42	0.87

NovelTSSR001188	G097	50	2	2.10	0.82	0.52	0.53	0.79
NovelTSSR001634	G109	50	3	2.87	0.84	0.51	0.44	0.72
	Mean	50	3.3	2.49	0.94	0.50	0.54	0.85
	St. Dev		0.96	0.47	0.20	0.11	0.12	0.08

* na = Observed number of alleles
* ne = Effective number of alleles [Kimura and Crow (1964)]
* I = Shannon's Information index [Lewontin (1972)]
# calculated using Power Marker Software

Table S12. Summary of heterozygosity statistics for all Loci.

Primer ID	Locus	Sample Size	Obs_Hom	Obs_Het	Exp_Hom*	Exp_Het*	Nei**	Ave_Het	Fis
C01P3AA00057	G002	50	0.18	0.82	0.37	0.62	0.62	0.62	-0.33
C01P3AA00134	G004	50	0.48	0.52	0.33	0.67	0.66	0.66	0.21
C01P6AA002298	G008	50	0.16	0.84	0.28	0.72	0.71	0.71	-0.18
C01P3AA002665	G009	50	0.16	0.84	0.43	0.57	0.57	0.57	-0.48
C01P2AA005299	G021	50	0.20	0.80	0.31	0.69	0.68	0.68	-0.17
A2M001208	G030	50	0.12	0.88	0.49	0.52	0.51	0.51	-0.73
A2M000167	G037	50	0.62	0.38	0.45	0.55	0.54	0.54	0.29
AB2M006344	G040	50	0.40	0.60	0.49	0.52	0.51	0.51	-0.18
AB2M006866	G041	50	0.76	0.24	0.49	0.51	0.51	0.51	0.53
AB2M002966	G052	50	0.16	0.84	0.37	0.63	0.63	0.63	-0.34
NovelTSSR000395	G063	50	0.16	0.84	0.28	0.72	0.71	0.71	-0.18
NovelTSSR000641	G075	50	0.12	0.88	0.49	0.52	0.51	0.51	-0.73
NovelTSSR000760	G084	50	0.16	0.84	0.28	0.72	0.71	0.71	-0.18
NovelTSSR001188	G097	50	0.16	0.84	0.25	0.75	0.71	0.71	-0.18
NovelTSSR001634	G109	50	0.48	0.52	0.33	0.67	0.66	0.66	0.21
	Mean	50	0.28	0.71	0.37	0.62	0.62	0.62	-0.17
	St. Dev		0.21	0.21	0.09	0.09	0.08	0.08	0.36

* Expected homozygosty and heterozygosity were computed using Levene (1949)

** Nei's (1973) expected heterozygosity
Wright's (1978) fixation index (Fis) as a measure of heterozygote deficiency or excess

Table S13. The Ewens-Watterson test for neutrality.

Primer ID	Locus	n	k	Obs. F	Min F	Max F	Mean*	SE*	L95*	U95*
C01P3AA00057	G002	50	3	0.383	0.333	0.961	0.678	0.032	0.376	0.961
C01P3AA00134	G004	50	3	0.340	0.333	0.961	0.678	0.033	0.362	0.961
C01P6AA002298	G008	50	6	0.291	0.167	0.905	0.426	0.022	0.227	0.796
C01P3AA002665	G009	50	3	0.433	0.333	0.961	0.668	0.033	0.370	0.961
C01P2AA005299	G021	50	4	0.317	0.250	0.942	0.563	0.031	0.303	0.904
A2M001208	G030	50	3	0.490	0.333	0.961	0.674	0.032	0.380	0.961
A2M000167	G037	50	3	0.459	0.333	0.961	0.674	0.033	0.362	0.961
AB2M006344	G040	50	3	0.491	0.333	0.961	0.672	0.033	0.365	0.961
AB2M006866	G041	50	3	0.494	0.333	0.961	0.674	0.032	0.371	0.961
AB2M002966	G052	50	4	0.372	0.250	0.942	0.562	0.028	0.310	0.922
NovelTSSR000395	G063	50	3	0.340	0.333	0.961	0.678	0.033	0.362	0.961
NovelTSSR000641	G075	50	4	0.317	0.250	0.942	0.563	0.031	0.303	0.904
NovelTSSR000760	G084	50	2	0.479	0.323	0.961	0.674	0.033	0.362	0.961
NovelTSSR001188	G097	50	2	0.469	0.313	0.961	0.674	0.033	0.362	0.961
NovelTSSR001634	G109	50	3	0.340	0.333	0.961	0.678	0.033	0.362	0.961

* These statistics were calculated using 1000 simulated samples.

Table S14. 2-way Mantel (1967) method test results.

Item		Value
N		1225
Mean X		0.3930
SSx		30.7357
Mean Y		0.3930
SSy		12.6846
Matrix correlation:	r	0.64242
Raw Mantel statistic	Z	201.9119
Approximate Mantel t-test:	t	12.3386
Prob. random Z < obs. Z:	p =	1.0000

Table S15. List of accessions used in this study.

A. Accessions used for primer test

Sl No	Code of accession	Name of accession	Genomic Background	Sl No	Code of accession	Name of accession	Genomic Background
Use for primer screening							
1	GY037	Prata	AAB	5	GY0109	FHIA 17	AAAA
2	GY0160	Kluai namwa khom	ABB	6	GY070	BITA2	ABBB

3	GY011 2	FHIA-21(#68)	AAAB	7	BGY3	BGY3	BB
4	GY060	Dwarf Cavendish	AAA	8	GY0127	FHIA-03	AABB

B. Accessions used for diversity study

Gel len	MGIS ID	Name	Genome	Sub group
1	GY0018	Laknau	AAB	subgr. Laknau
2	GY0152	Chuoi Xi Mon	AB	subgr. Mysore
3	GY0075	Pisang Rajah (South Johnstone)	AAB	subgr. Pisang Raja
4	GY0090	Curare	AAB	subgr. Plantain
5	GY0083	GC6	AAA	Unknown
6	GY0103	Figue Pomme Géante	AAB	subgr. Silk
7	GY0072	Vunamami	AS	Unknown
8	GY0109	FHIA 17	AAAA	Unknown
9	GY0134	Rukumamb	AAB	Unknown
10	GY0019	TMP2x 2829-62	AA	Unknown
11	GY0021	IRFA 905	AA	Unknown
12	GY0030	Tuu Gia	AA	Unknown
13	GY0135	Luba	AAB	subgr. Iholena
14	GY0092	Simili Radjah	ABB	subgr. Peyan
15	GY0102	Dole	ABB	subgr. Bluggoe
16	GY0031	Pisang Jaran	AA	Unknown
17	GY0156	Formosana	AAA	subgr. Cavendish
18	GY0117	Intokatoke	AAA	subgr. Mutika/Lujugira
19	GY0126	Lai	AAA	subgr. Red
20	GY0049	Pisang Berlin	AA	Unknown
21	GY0099	NBB 11	AA	Unknown
22	GY0001	Obubit Ntanga green mutant	AAB	subgr. Plantain
23	GY0006	Akpakpak	AAB	subgr. Plantain
24	GY0007	Orishele	AAB	subgr. Plantain
25	GY0101	Pa (Rayong)	AA	subsp. siamea
26	GY0137	Utafun	Fe'i	Fe'i
27	GY0143	Asupina	Fe'i	Fe'i
28	GY0146	Menei	Fe'i	Fe'i
29	GY0033	SH 3436-6	?	Unknown
30	GY0004	Ibwi	AAA	Unknown
31	GY0106	Pisang Lilin	AA	Unknown
32	GY0122	Sowmuk	AA	Unknown
33	GY0008	Kazirakwe	AAA	subgr. Mutika/Lujugira
34	GY0009	Williams (Bell, South Johnstone)	AAA	subgr. Cavendish
35	GY0010	Igitsiri (Intuntu)	AAA	subgr. Mutika/Lujugira
36	GY0013	Mbwazirume Nakitembe cooking	AAA	subgr. Mutika/Lujugira

		Ingagara Nakitembe		
37	GY0014	cooking	AAA	subgr. Mutika/Lujugira
38	GY0035	Highgate	AAA	subgr. Gros Michel
39	GY0060	Dwarf Cavendish	AAA	subgr. Cavendish
40	GY0071	Igitsiri (Intuntu)	AAA	subgr. Mutika/Lujugira
41	GY0082	Leite	AAA	subgr. Rio
42	GY0086	Nyamwihogora	AAA	subgr. Mutika/Lujugira
43	GY0088	Pisang Bakar	AAA	subgr. Ambon
44	GY0095	Chuoi Tieu cao hong	AAA	subgr. Cavendish
45	GY0100	Petite Naine	AAA	subgr. Cavendish
46	GY0113	GC7	AAA	Unknown
47	GY0141	Khai Nai On	AA	Unknown
48	GY0160	Kluai namwa khom	ABB	Unknown
49	GY0154	Ensete ventricosum	ENSETE	ENSETE
50	GY0128	Dwarf Parfitt	AAA	subgr. Cavendish

Code of accession based on Musa Germplasm Information System (https://www.crop-diversity.org/mgis/accession-search?f%5B0%5D=collection%3AIFTR/GDAAS)

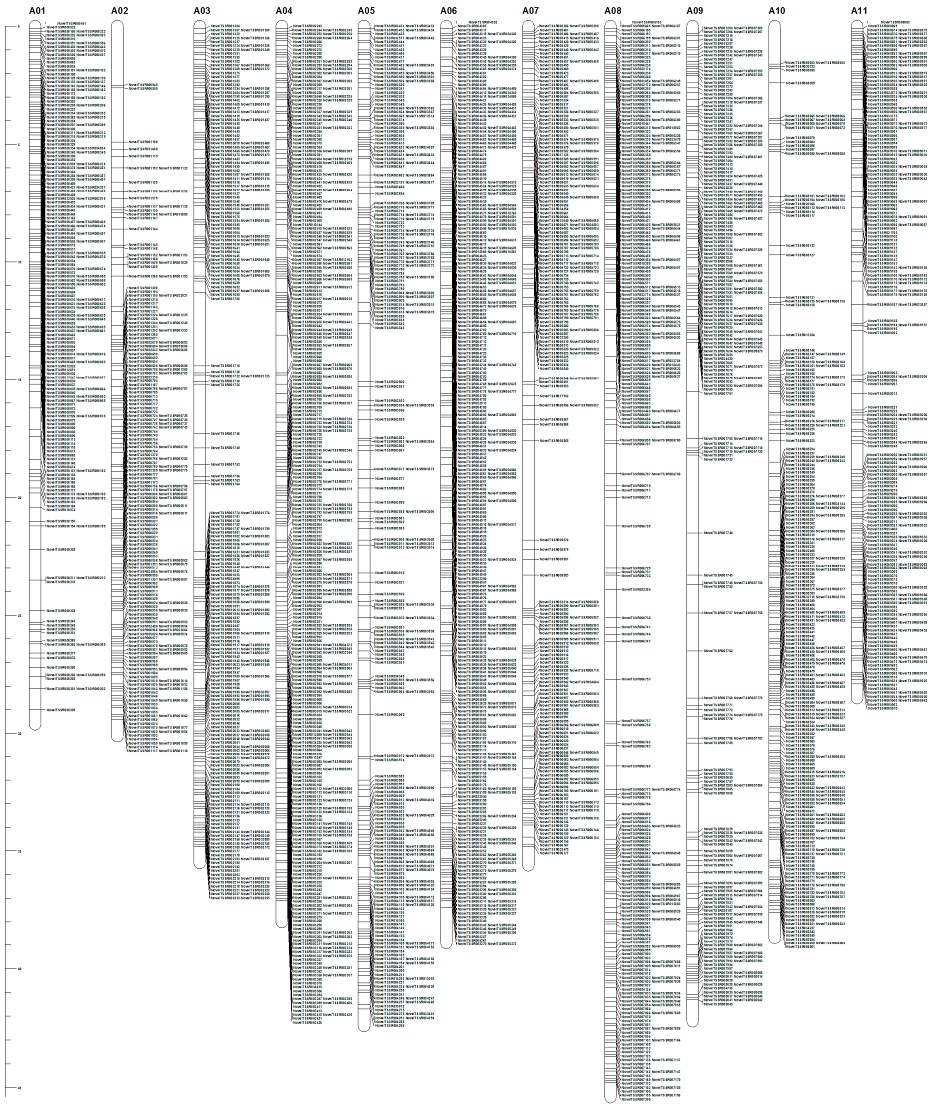

Figure S1. Physical map of *M. acuminata* genome specific FRSMs. A genomic distribution of 2871 functionally relevant SSRs marker on the eleven chromosomes of *M. acuminata*. Left bar represents chromosome length (Mb).

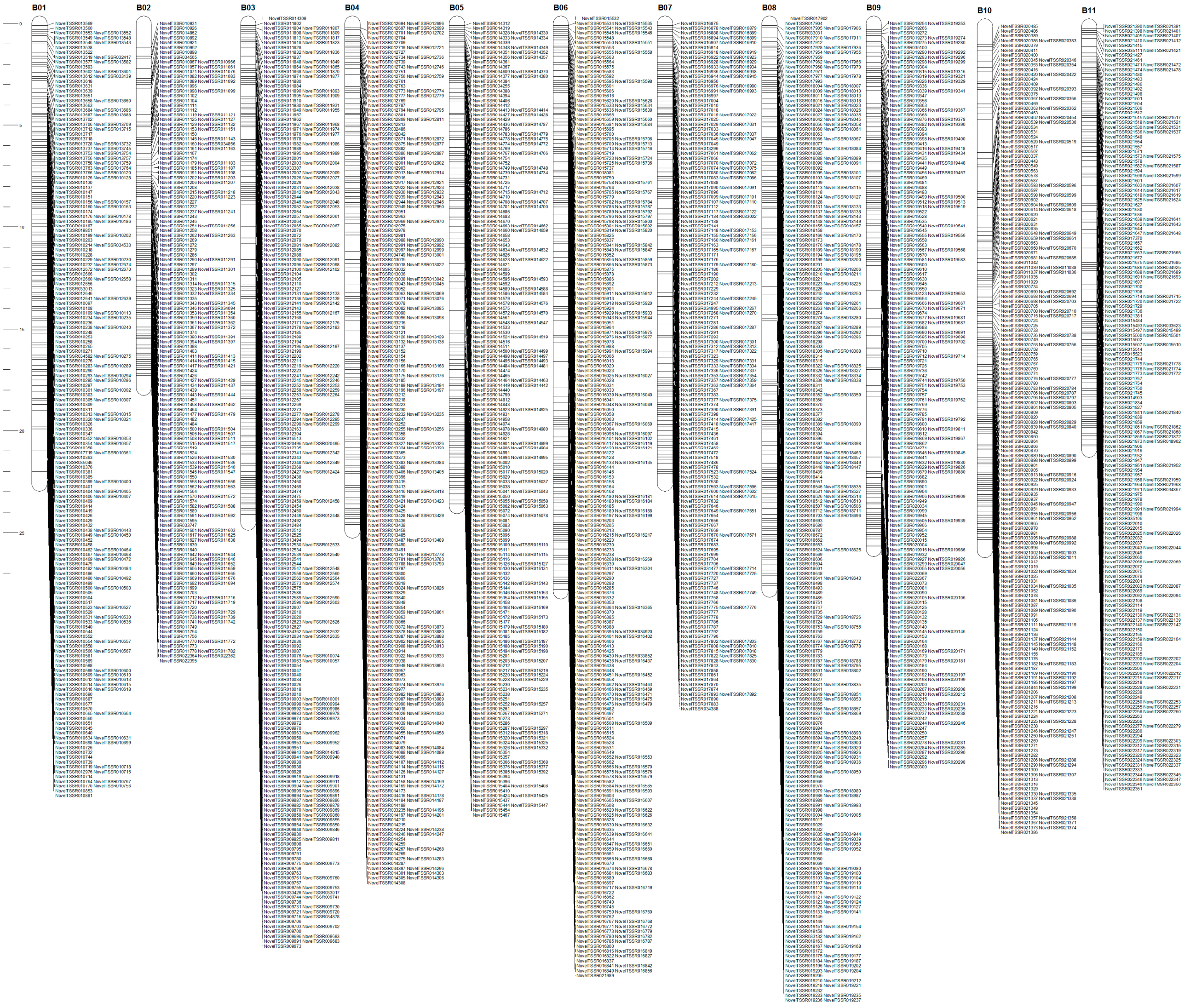

Figure S2. Physical map of *M. balbisiana* genome specific FRSMs. A genomic distribution of 2754 functionally relevant SSR markers on the eleven chromosomes of *M. balbisiana*. Left bar represents chromosome length (Mb).

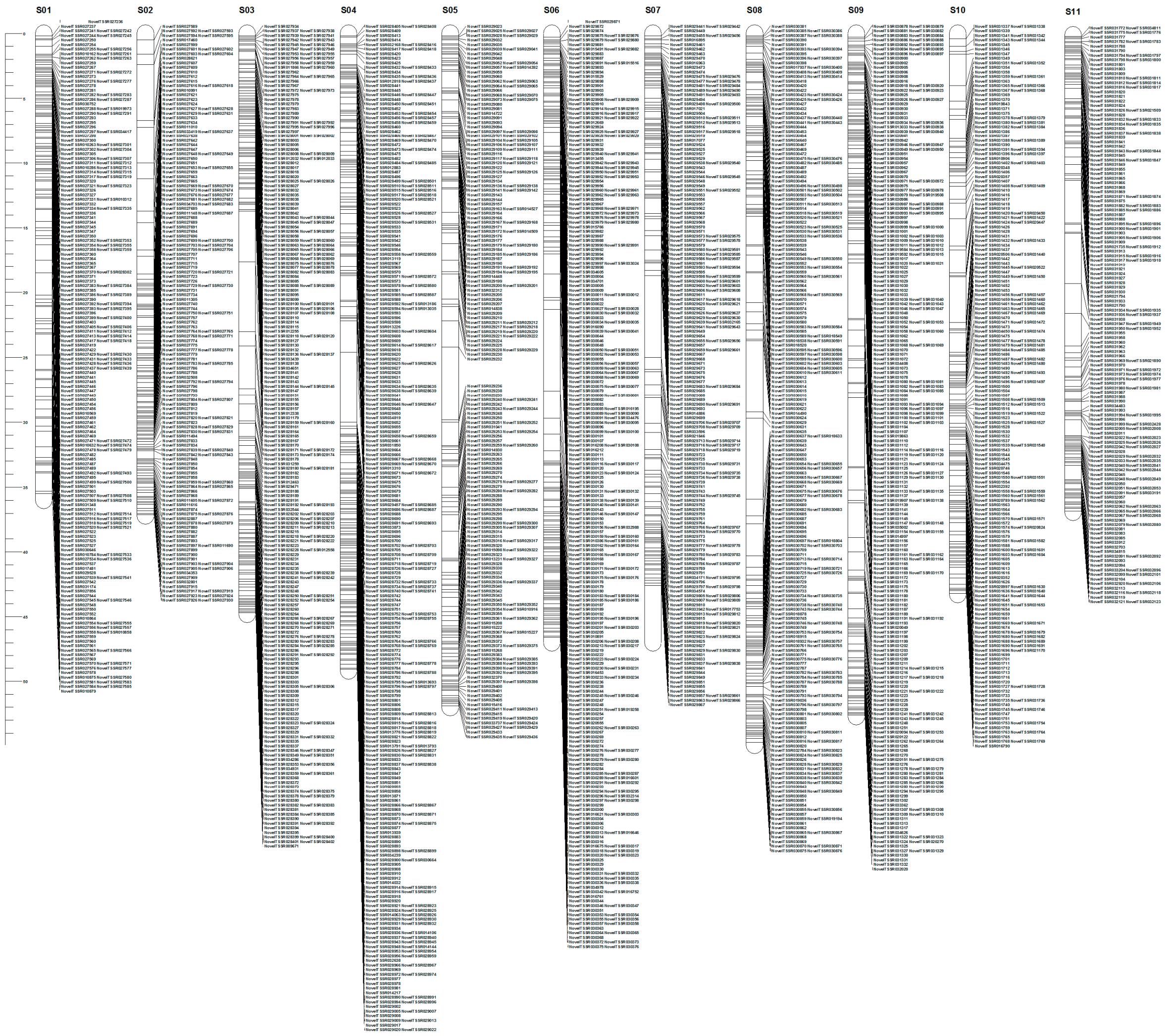

Figure S3. Physical map of *M. schizocarpa* genome specific FRSMs. A genomic distribution of 2591 functionally relevant SSR markers on the eleven chromosomes of *M. schizocarpa*. Left bar represents chromosome length (Mb).

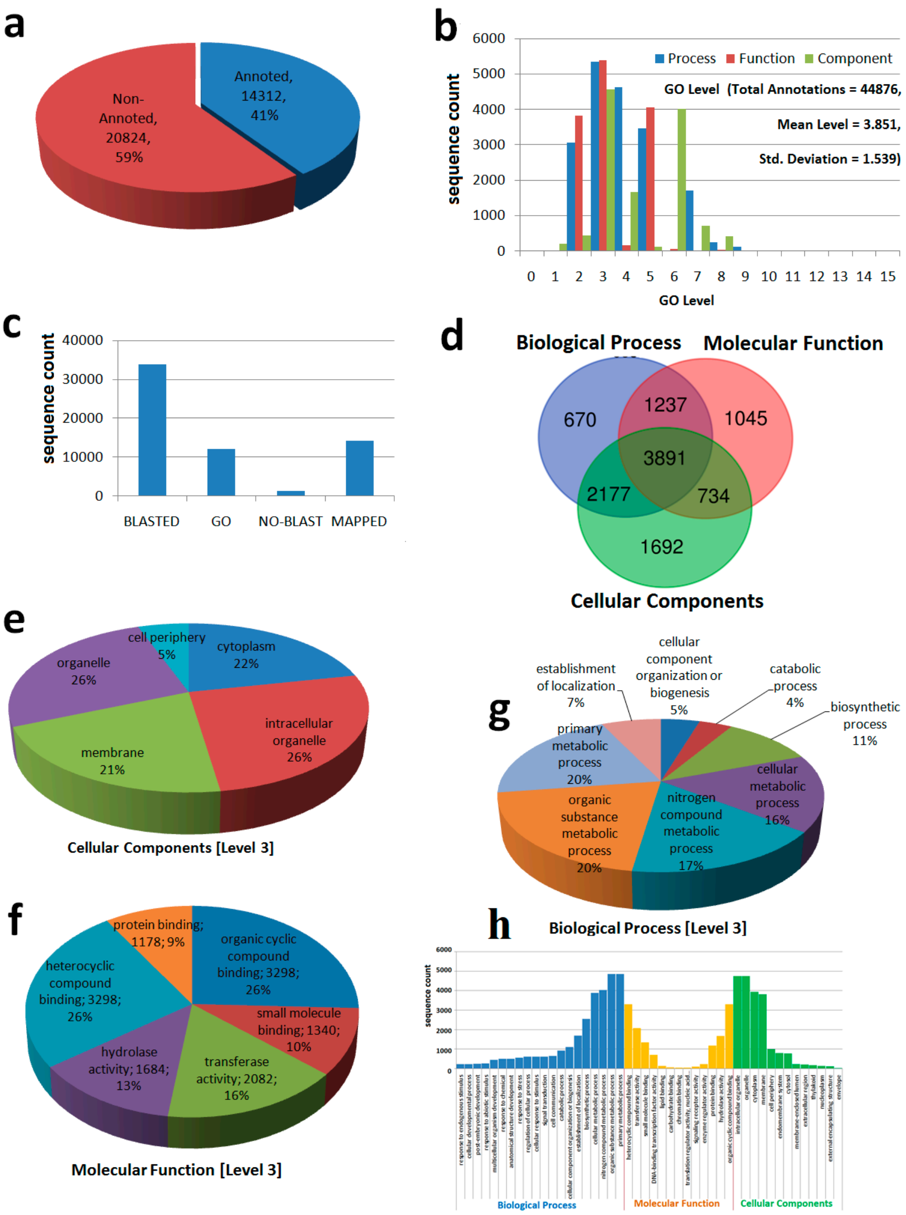

Figure S4. Functional annotation and GO classification of FRSM markers. Distribution of FRSM markers among three GO categories. Top 20 GO terms of the GO Distribution.

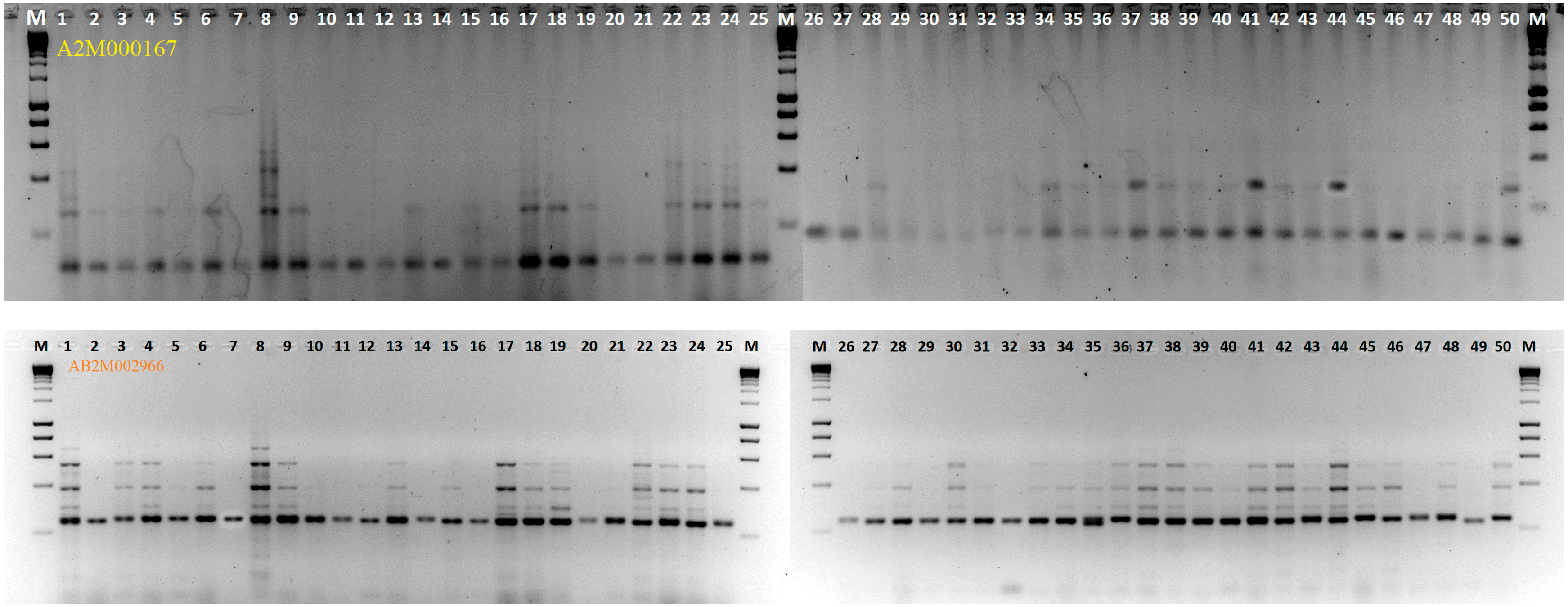

Figure S5. Allelic variation represent by FRSM markers (Marker: A2M000167 and AB2M002966). Len 1 to 50 represent the genotypes (Name of the genotype and genomic composition listed in Table S15) and M=100bp Marker. .

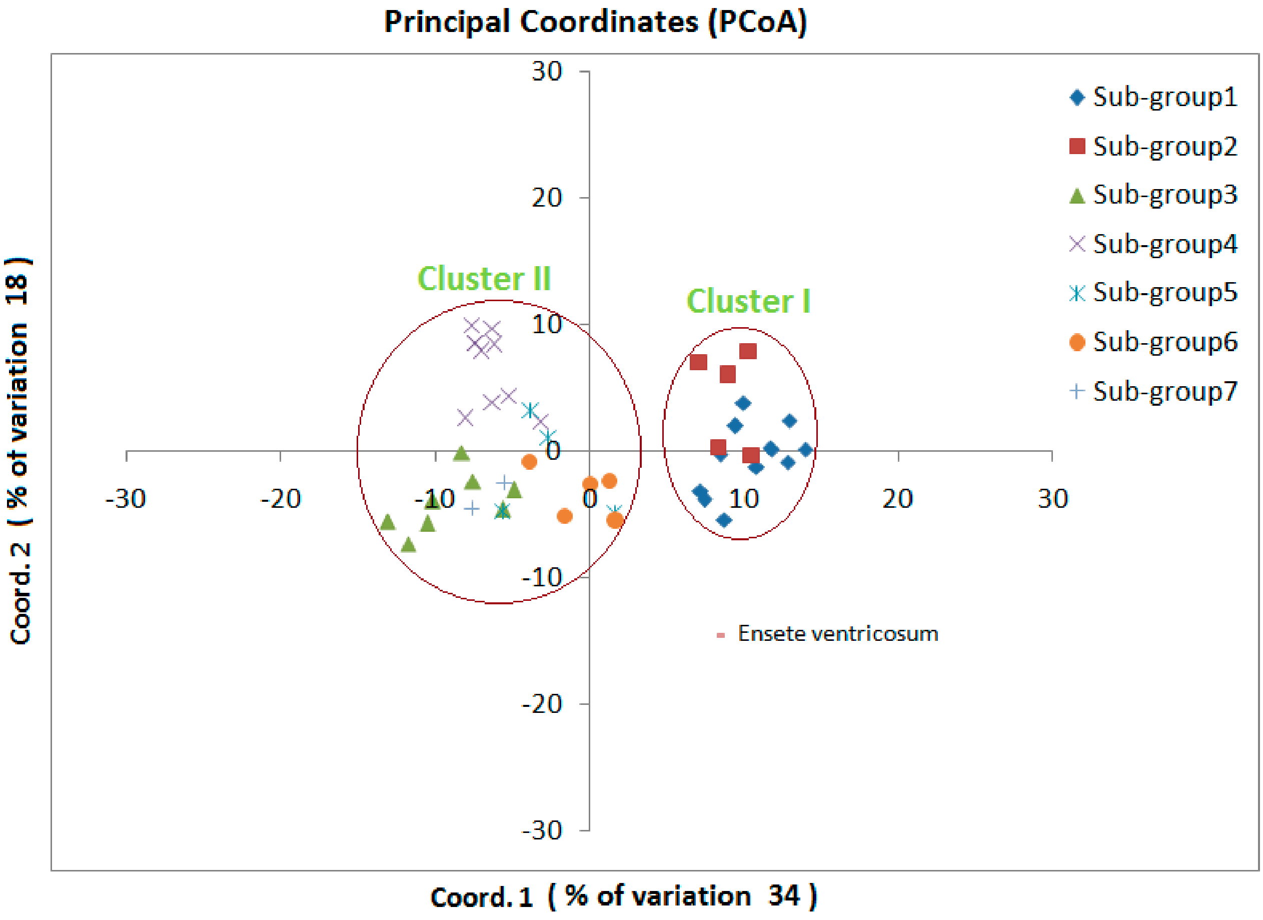

Figure S6. Principal coordinate analysis (PCoA) of 50 individuals based on genotypic information from 49 alleles obtained from 15 SSR markers. Here Cluster I represent AB genomic composition genotypes and Cluster II represent genotypes with only the A genome.
